# Temperature dependence of liverwort diversification reveals a cool origin and hot hotspots

**DOI:** 10.1038/s41598-025-87206-1

**Published:** 2025-01-25

**Authors:** Karola Maul, S. Robbert Gradstein, Dietmar Quandt, Michael Kessler

**Affiliations:** 1 Bonn Institute of Organismic Biology (BIOB), University of Bonn, Bonn, Germany; 2 Meise Botanic Garden, 1860 Meise, Belgium; 3 Department of Systematic and Evolutionary Botany, University of Zurich, Zurich, Switzerland

**Keywords:** Plant ecology, Speciation, Biodiversity

## Abstract

The evolutionary history underlying gradients in species richness is still subject to discussions and understanding the past niche evolution might be crucial in estimating the potential of taxa to adapt to changing environmental conditions. In this study we intend to contribute to elucidation of the evolutionary history of liverwort species richness distributions along elevational gradients at a global scale. For this purpose, we linked a comprehensive data set of genus occurrences on mountains worldwide with a time-calibrated phylogeny of liverworts and estimated mean diversification rates (DivElev) and mean ages (AgeElev) of the respective genera per elevational band. In addition, we reconstructed the ancestral temperature preferences of the genera. We found that diversification rates increase linearly with temperature, and hence decrease with elevation. This pattern is mainly driven by epiphytic genera. In contrast, overall genus age is highest at intermediate elevations where liverwort species richness peaks and decreases towards both ends of the elevational and thermal gradient. Our results further indicate that the ancestral lineages from which the extant liverwort genera descended had a preference for cool and humid habitats. We conclude that the extant liverwort species diversity accumulated over long time under these climatic conditions, which are today prevailing at mid-elevations of the world’s mountains. Subsequently, liverworts expanded their ranges from these temperate areas towards warm (with high diversification rates) and cold regions (with low diversification rates), located in contemporaneous (tropical) lowlands and high mountains, respectively. The conserved preference for temperate climates shared by the majority of liverwort lineages gives reason to the assumption that they will not be able to cope with the conditions induced by rapid climate warming, whereas the current low-elevation radiation may be less affected by climate change.

## Introduction

Understanding the mechanisms behind the geographical variation of species richness is a major goal of ecology, and crucial for the understanding of natural life on earth and its conservation. The link between species richness and speciation seems intuitively self-evident: the more species originate, the more species will accumulate over time. This simple equation includes three factors: time, speciation, and extinction. Unfortunately, the magnitude of extinction of organisms is often difficult to ascertain, because unless fossils are present, we can only indirectly infer the past existence of extinct lineages^1,2^. Therefore, ecologists commonly employ net diversification rates calculated as speciation rate ($\lambda$) minus extinction rate $(\mu )$ as an approximation^3^.

Several partly conflicting hypotheses have been proposed on how time (lineage age) and diversification rates interact to shape spatial gradients of biodiversity: The time-for-speciation effect affects different ecological processes like adaptation and extinction, and thus allows species accumulation with the passage of time^4^. To generate gradients in species richness, this effect is often combined with the concept of niche conservatism, in which a lineage has to develop novel adaptations to be able to survive in habitats different from its ancestral ecological niche, thus resulting in a time lag and a diversity gradient away from the habitat of origination^5^. Together, the concepts of time-for-speciation and niche conservatism propose that a group of organisms will be most diverse under the environmental conditions of the niche at which it originated and has accumulated high richness of species over time^5,6^. For instance, Stevens^7^ found that the latitudinal gradient in species richness distribution of New World phyllostomid bats is linked to both, the spatial characteristics of the time for speciation hypothesis and the environmental characteristics associated with tropical niche conservatism. On the other hand, however, the reverse pattern may also be found: as a group of organisms expands its ecological amplitude, adaptive radiations in novel habitats may lead to high diversification rates (speciation exceeds extinction by far). Accelerated diversification then may spatially overlap with high species richness^8^ or proceed in comparatively species poor habitats^9^. Indeed, recent research suggests that there is no universal relationship between diversification rates and species richness patterns, and that time-for-speciation and ecological processes are more important instead^10,11^.

Species accumulation may also be influenced by environmental conditions. For instance, the Metabolic Theory of Ecology proposes that warm, wet climates lead to high metabolic rates as well as high population densities of species, and that these together favor speciation and lower extinction rates^12–14^. The same effect may be the result of climatic stability in the past, which may also reduce extinction rates^15^, although the opposite effect—increased speciation rates associated with earlier climatic instability—has also been found^16^. On the other hand, recent seasonal climatic variability requires species to have broad climatic niches, reducing the ability for fine-tuned niche segregation^17,18^. Opposed to this, high habitat heterogeneity will likely increase speciation rates because it offers ample opportunities for niche segregation and the island-like distribution of habitats favors allopatric speciation^19,20^, even though small, scattered populations may also suffer from high extinction rates^21^. Finally, certain ecoregions may have fundamental characteristics that favor high diversity, such as historically large surface areas (e.g. Amazonia^22^) or a dynamic history (e.g. mountains^23,24^) .

Diversification rates may further be promoted or inhibited by biotic factors including the interactions between competitors, hosts and parasites / epiphytes, and plants and pollinators or seed dispersers^25^. For example, competition between clades has been found to limit diversification in canids^26^ and catfish^27^. Aguilée et al.^28^ suggest that the influence of competition for resources depends on the phase during the process of diversification, with an initial adaptive radiation characterized by low interspecific competition and great impact of resource abundance. During the second phase, termed “niche self-structuring”, interspecific competition leads to increased resource specialization of species, a dynamic which accelerates diversification rates. In the final stationary phase, species richness is more or less stable under the influence of strong interspecific competition, slowing down speciation rates. Importantly, along an ecological or geographical gradient, assemblages may be at different phases of this progress, depending on the time passed since each part of the gradient was colonized.

Mountains are global centers of biodiversity^29^. This applies to plants as a whole, but is especially pronounced in bryophytes and ferns, whose local and regional diversity peaks in the cloud forests of tropical mountains^30–34^. Because mountains are associated with several characteristics which potentially promote increased diversification (strong climatic gradients in combination with a dynamic geological history and high topographical complexity), they are also commonly considered to be centers of biological diversification^35–39^. For these reasons, it has been proposed that current diversification rates might increase with increasing elevation, as indeed found in a global study of birds^9^. In contrast, based on time-for-speciation together with the out-of-the-tropics hypothesis^40,41^ clade ages might be expected to decrease towards higher elevations, since ancestral niches are commonly considered to correspond to tropical lowland conditions^42^. This was indeed found for ferns in Japan^43^ but not for woody angiosperms in tropical South America and in tropical Asia^44^, suggesting that time-for-speciation does not show a consistent elevational pattern.

Liverworts are one of the earliest diverging extant land plant lineages, with the oldest fossil undoubtedly belonging to liverworts being from the Pragian (407–411 Ma)^45^ and an estimated origin of bryophytes in the Cambrian/Ordovician (506.4–460.3 Ma)^46^ and of liverworts in the Cambrian (536–516 Ma)^47^. There are about 5000–6000 extant liverwort species^48^ which inhabit almost every biome on the land surface of the planet, ranging from the coast of Antarctica to tropical forests and non-forested habitats including tundra, steppes, and freshwater systems^49^. Correspondingly, liverworts colonize a large variety of microhabitats and grow on soil, rocks and rotting wood, as well as on different parts of other living plants, e.g., roots, tree trunks, branches and even leaves. Thus, liverwort species can roughly be classified as either non-epiphytes, facultative epiphytes, or obligate epiphytes^50^. Generally speaking, phylogenetically basal liverwort lineages (Haplomitriopsida), complex thalloid liverworts (Marchantiopsida), and simple thalloids I (Pelliidae) contain non-epiphytic species, whereas simple thalloids II (Metzgeriidae) plus leafy liverworts (Jungermanniidae) comprise all the three lifestyles^51,52^. Many obligate epiphytes are members of the Porellales order and show characteristic adaptations to their preferred substrate, while the majority of Jungermanniales species are generalists that have apparently not evolved specific adaptations to epiphytism^53^. Despite the ubiquitous occurrence of liverworts in general, most species prefer cool and humid conditions, found around mid-elevations in mountains, which are the global hotspots of liverwort species richness^54^.

The diversification history of liverworts has been subject of earlier studies, focusing on particular clades or geographic regions, the variability of diversification rates over time, and the causes underlying the latitudinal gradient in species richness: The first major study on the subject by Wilson et al.^55^ found constant diversification in the Lejeuneaceae (Porellales) during the Cenozoic after rapid initial divergence during the late Cretaceous. Subsequently, Feldberg et al.^52^ investigated diversification of leafy liverworts and found epiphytism in Porellales to be linked to increased speciation rate during the Cretaceous as opposed to rather constant diversification rates in the generalistic Jungermanniales. Silva et al.^56^ found evidence for increased species accumulation in the genus *Frullania* (Frullaniaceae, Porellales) during the Paleogene and Neogene. The findings of a study on the distribution of diversification rates of the bryophyte flora of China by X. Song et al.^57^ indicate the positive relationship between liverwort diversification rates and high temperature, as well as the availability of water, and the elevational extent. Laenen et al.^47^ found evidence for increased diversification rates since the mid-Mesozoic and Cenozoic in bryophyte lineages, and conclude that the low extant diversity of bryophytes compared to angiosperms also results from substantial extinctions in the past. Laenen et al.^42^ observed significantly higher rates of net diversification in tropical liverwort genera than in non-tropical ones and suggest this to be causal for the latitudinal gradient in liverwort species richness.

Recently, Qian et al.^58^ found that the phylogenetic diversity of the liverwort flora of China increased with latitude in contrast to species richness. This is interpreted to indicate a non-tropical origin of the extant liverwort taxa. Accordingly, Qian and Kessler^59^ analyzed the species richness distribution of an elevational gradient in the Andes and observed the highest species richness at mid-elevations, but the maximum phylogenetic diversity was located at higher elevations.

Despite these studies, the relationship of diversity patterns of liverworts with elevation in relation to the underlying evolutionary history remains unexplored.

In this study we therefore aimed to shed further light on the evolutionary history of liverworts and their worldwide elevational diversity patterns by addressing the following questions:

1. What are the elevational distribution patterns of diversification rates and genus age in liverworts?
2. Do these patterns differ between epiphytic and non-epiphytic liverworts?
3. Do these patterns allow for inferences on the evolution of temperature preferences in liverworts?

## Materials and methods

### Data set

**Fig. 1:**
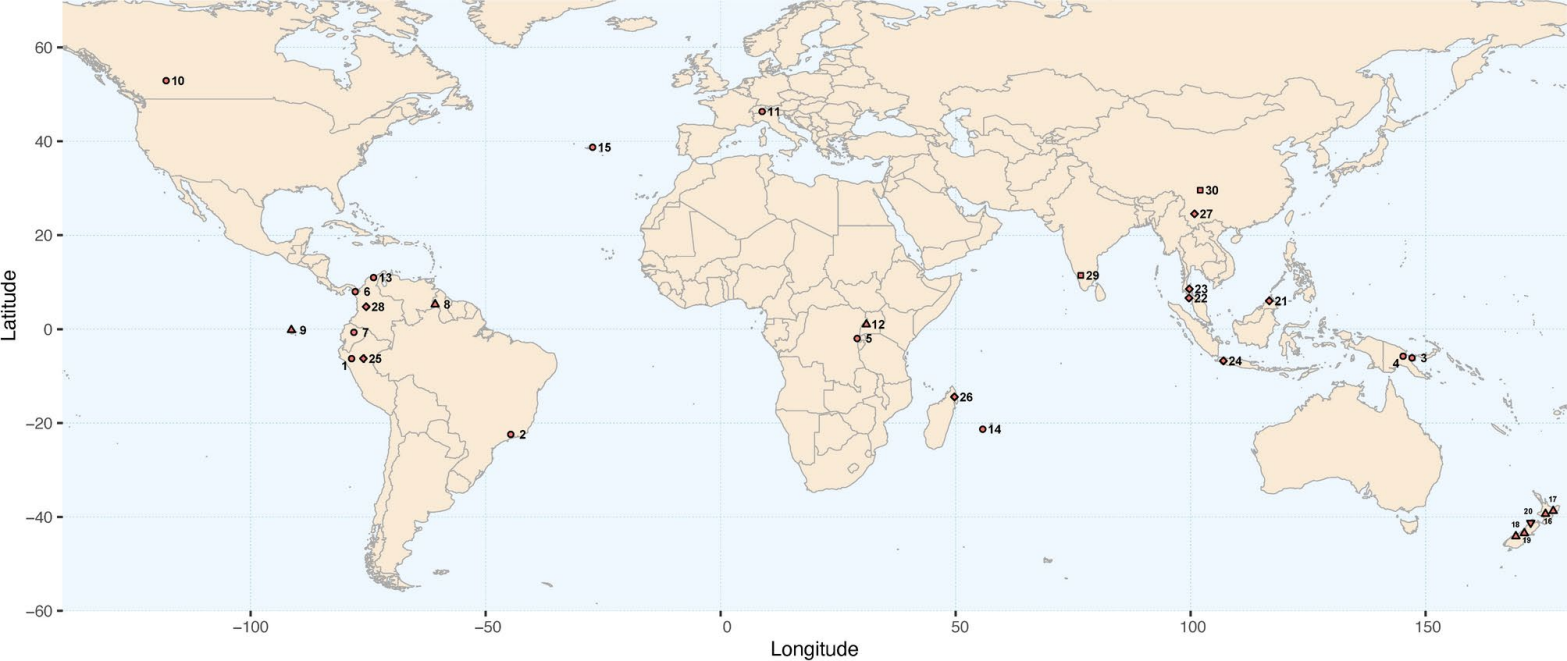
Global map illustrating geographical situations of elevational gradients analyzed in this study. Numbers correspond to Table 1, red icons represent life-forms of liverworts recorded from the individual gradients: all (circles), epiphytic (diamonds), non-epiphytic (squares), all + epiphytic + non-epiphytic (triangles), all + epiphytic (upside-down triangle). The map was drawn using the R package “ggplot2” 3.3.3.^88^.

We compiled a dataset containing 17,994 genus records from 26 publications (Table 1, Fig. 1). The studies considered the following conditions: (1) they comprised at least four elevational bands (or could be transformed into such), (2) the source data contained a minimum of 10 taxa, and (3) the study terrain roughly corresponded to the successive reduction of land surface area with elevation^60^. Because we intended to analyze the liverwort community at a regional scale, elevational species ranges were sometimes taken from broader, regional works. Thus, elevational distribution of species in the source publications may have referred to a wider geographical area. For instance, data of elevational species distribution recorded from Napo province, Ecuador, which were extracted from the Ecuadorian checklist^61^, originally referred to the wider northern Andean region and were subsequently corrected for the species with the lowest elevational records beyond Napo, based on observations of one of the authors. The generic taxonomy of all species records were updated based on Söderström et al.^62^. We likewise updated the taxonomy of the Liverwort Tree of Life provided in Laenen et al.^47^. The taxon sampling of Laenen et al.^44^ covered about 84% of generic diversity of liverworts, represented by 303 taxa. The phylogenetic analyses are based on eight genetic markers of chloroplast, mitochondrial and nuclear DNA. The authors thoroughly resolved topological conflicts between the individual marker alignments and used 25 fossils to time-calibrate the phylogeny (for more details see methods in^47^ and the calibration I scheme, respectively).

**Table 1:** Properties of source data included in this study (A, All; E, Epiphytes; NE, Non-Epiphytes; NP, National Park; DRC, Democratic Republic of the Congo; modified after Maul et al.^54^).

No	Project/Expedition/Publication	Life forms	Study method	Location, Country	Elevational sampling range (m)	Number of elevational steps	Upper gradient limit (m)
1	Bryotrop I Expedition, Schultze-Motel and Menzel^63^	A	transect plots	Andes, Northeastern Peru	200–3400	17	4000
2	da Costa et al.^64^	A	herbarium survey	Itatiaia NP, Brazil	600–2700	10	2787
3	Koponen-Norris Expedition, Enroth^65^, Supplementary Appendix 1	A	targeted searches	Huon Peninsula, Papua New Guinea	0–3700	34	4000
4	3 transects Expedition 2nd part, Maul et al.^54^	A	transect plots	Mt. Wilhelm, Papua New Guinea	200–3700	8	4459
5	Bryotrop III Expedition, Fischer^66^, Supplementary Appendix 1	A	targeted searches	Kahuzi-Biega-NP, DRC/ Nyungwe Forest, Virungas, Rwanda	850–4507	27	4507
6	Gradstein and Salazar-Allen^67^	A	transect plots	Darién NP, Panama	50–1150	5	1435
7	Gradstein^61^	A	checklist	Napo, Ecuador	300–4800	46	4800
8	Gradstein and Florschütz-de Waard^68^	A, E, NE	targeted searches	Mt. Roraima, Guyana	500–2300	14	2400
9	Gradstein and Weber^69^	A, E, NE	herbarium survey + targeted searches	Galapagos Islands, Ecuador	10–1100	6	1300
10	Lee^70^	A	transect plots	Jasper NP, Canada	1067–2227	14	2694
11	Swissbryophytes^71^	A	checklist	Switzerland	600- 3000	8	3320
12	3 transects Expedition 1st part, Maul et al.^72^	A, E, NE	transect plots	South-Western Uganda	680–3200	24/24/23	3645
13	van Reenen et al.^73^	A	transect plots	Sierra de Santa Marta, Colombia	500–4100	19	4500
14	Ah-Peng et al.^74^	A	transect plots	Piton des Neiges, La Réunion	250–850	4	3070
15	Coelho et al.^75^	A	transect plots	Pico Island, Portugal	10–2200	12	2350
16	Lindlar and Frahm^76^ + Pfeiffer^77^	A, E, NE	transect plots	Mt. Ruapehu, New Zealand	650–1450	5	2400
17	Lindlar and Frahm^76^ + Pfeiffer^77^	A, E, NE	transect plots	Urewera NP, New Zealand	600–1150	5	1392
18	Lindlar and Frahm^76^ + Pfeiffer^77^	A, E, NE	transect plots	Haast Pass, New Zealand	100–800	5	2000
19	Lindlar and Frahm^76^ + Pfeiffer ^77^	A, E, NE	transect plots	Franz-Josef Glacier, New Zealand	15–800	7	1800
20	Lindlar and Frahm^76^ + Pfeiffer^77^	A, E	transect plots	Karamea, New Zealand	20–1000	6	1875
21	Bryotrop II Expedition, Kürschner^78^	E	transect plots	Mt. Kinabalu, Malaysia	50–3500	18	4095
22	Chantanaorrapint^79^	E	transect plots	Tarutao Island, Thailand	25–700	4	700
23	Chantanaorrapint^80^	E	transect plots	Khao Luang, Khao Nan, Thailand	400–1550	8	1780
24	Iskandar et al.^81^	E	transect plots	Mt. Gede, Indonesia	1500–2700	7	3100
25	BRYOSTRAT Project, Kürschner and Parolly^82^	E	transect plots	Andes, Northeastern Peru	280–3300	17	4000
26	Marline^83^	E	transect plots	Marojejy NP, Madagascar	250–2050	10	2132
27	L. Song et al.^84^	E	transect plots	Mengla, Zhenyuan, Lijiang, China	800–3800	12	4000
28	Wolf^85^	E	transect plots	Cordillera Central, Andes, Colombia	1000–4130	15	4150
29	Alam^86^	NE	herbarium survey + targeted searches	Nilgiri Hills, India	1100–2600	16	2623
30	Sun et al.^87^	NE	transect plots	Gongga Mountains, China	2300–4220	12	4900

We subsequently reduced synonymous species or genera to one tree tip of this chronogram, taxa with a doubtful position in the phylogeny were pruned (*Chandonanthus, Leptolejeunea*), and non-monophyletic clades were lumped together (e.g., *Colura*, *Myriocoleopsis*). 174 genera from our dataset could be assigned to the genera of the phylogenetic tree, 37 genera of our dataset (3.81% of the genus per elevation records) were not assignable and were thus excluded from the analyses (Supplementary Table S2). We assigned records of the genus *Lophocolea* to the *Chiloscyphus* sp. tip in the phylogenetic tree, based on the study of Patzak et al.^89^. To estimate the reliability of the phylogeny we ran 1,000 bootstrap replicates^90^ (ML criterion, GTR + Γ + I substitution model) using the alignment of Laenen et al.^47^ which we pruned according to our data set (Supplementary Fig. S1).

### Diversification rates and genus ages

We calculated the maximum likelihood diversification rates for each genus after Magallón and Sanderson^3^ as $\widehat{r}$= log (n)/t. Species numbers per genus (n) were taken from Laenen et al.^47^, and updated by Gradstein^48^, Lee and Gradstein^91^, and additional unpublished information (Supplementary Table S1). Stem lineage ages (t) were taken from Laenen et al.^47^. Subsequently, we calculated the arithmetic means of genus diversification rates and genus age per elevational band (DivElev, AgeElev) for each elevational gradient of our dataset. A one-way ANOVA was performed to compare the DivElev and AgeElev between the groups of overall, epiphytic, and non-epiphytic liverwort datasets. In case of significant ANOVA result, a Tukey’s HSD Test was used for an exact differentiation between the three groups.

### Climatic variables

We obtained layers of Bioclim data (temperature: annual mean (Bio1), max of warmest month (Bio5), min of coldest month (Bio6), and precipitation amount: annual (Bio12), of wettest month (Bio13), of driest month (Bio14), of warmest quarter (Bio18), and of coldest quarter (Bio19)) from CHELSA^92^. We then calculated the climate data for our dataset by fitting linear models for temperature data, and linear or polynomial models (quadratic or cubic) for the precipitation amounts using coordinates at the approximate locations of the original plots and the respective elevations per gradient in cases where detailed geographical coordinates were unavailable. Only predicted precipitation variables from models with (adjusted) R^2^ > 0.5 were included in our dataset. We included the precipitation data referring to the exact location (without employing models) for those study transects with available GPS coordinates (Uganda, Madagascar, and Mt. Wilhelm in Papua New Guinea). A more detailed explanation of our approach for assigning climate data to our data set is provided in Maul et al.^54^.

### Regression analyses

Aiming to identify the elevational patterns of diversification rates and to draw conclusions about their drivers, we regressed the mean genus diversification rates per elevational band (DivElev) for the dataset of all (without specified habitat, 276 genus x elevation records from 20 gradients), epiphytic (163 genus x elevation records from 16 gradients), and non-epiphytic genera (90 genus x elevation records from 9 gradients) against elevation (Elev), relative elevation (relElev, absolute elevation as percentage of the maximum elevation the liverworts can occur to on the respective gradient), species richness (Rich), relative species richness (relRich, species richness as percentage of the maximum species richness found on the respective gradient), and the climatic variables using beta regression models. We tested the respective relationships for first and second order polynomial relationships. To assess the goodness of model fit we used the pseudo-R^2^ (${\text{R}}_{\text{p}}^{2}$) function suggested by Ferrari and Cribari-Neto^93^ implemented in the R package betareg 3.1.-4^94^. Likelihood ratio tests of nested models were conducted to decide if the linear or polynomial relationship was preferable.

Furthermore, we calculated the mean genus age per elevational band (AgeElev) of all, epiphytic, and non-epiphytic genera, and tested its relationship with the same set of independent variables with generalized linear models (GLMs) with Poisson distribution. To test the improvement of significance of the more complex model, the corrected Akaike Criterion AICc ^95^ was taken as reference. The variance explained by the models was assessed using the Kullback–Leibler-divergence-based R^2^^96^.

We repeated all analyses after reducing the dataset to one record per genus per elevational step (8,057 genus records in total) on each gradient to assess if the elevational pattern was driven by the accumulation of genera with exceptional high (or low) diversification rates or genus ages. Because geographical barriers may influence speciation and species assemblage of organisms on young islands where the newly arrived species are at the early stage of colonization and adaptation, the average age of genera on islands may differ from continental regions. We thus repeated the regression analyses after removing data from Galapagos Islands, La Reunion, and Pico Island.

### Ancestral state reconstruction

We calculated the arithmetic mean of the annual mean temperature (Bio1) and minimum temperature of the coldest month (Bio6) for each genus in our dataset based on the records along the transects and the corresponding climatic data. The tips of the chronogram were reduced to the genera occurring on the elevational gradients. First, we used Pagel’s lambda to infer the degree to which the distribution of temperature preference is dependent on phylogenesis^97^. Subsequently, the maximum likelihood of the ancestral mean temperature (Bio1) was estimated.

All computational analyses were conducted in R 4.0.2^98^ with the package “phangorn” 2.5.5^99^ for the bootstrap analysis, “raster” 3.4–5^100^ for the extraction of the bioclim data, “betareg” 3.1.-4^94^ to fit the beta regression models, “lmtest” 0.9–38^101^ to conduct likelihood ratio tests of nested models, “ape” 5.5^102^ to prune the phylogeny, “phytools” 1.0–1^103^ for the ancestral character reconstruction using the fastAnc function and its visualization, “MuMIn” 1.43.17^104^ for the calculation of AICc, and “rsq”^105^ to infer the goodness of fit of the GLMs.

## Results

### Diversification rates

The net diversification rates calculated for the 174 genera in our dataset ranged between 0 (in the 24 monospecific genera) and 0.69 speciation events per million years (mean = 0.07, median = 0.05; Supplementary Table S1). The majority 65.5%) of the studied genera had diversification rates between 0 and 0.1.

Diversification rates < 0.1 were scattered across the phylogeny with the exception of the Lejeuneaceae family, where accelerated rates accumulated. A total of 27 (15.5%) genera had diversification rates of 0.1–0.2, 15 (55.6%) of which were members of the family Lejeuneaceae. Nine genera were found to have diversification rates higher than 0.2: five from Lejeuneaceae, one from Lepidoziaceae, and three were from Cephaloziellaceae. *Acanthocoleus* (Lejeuneaceae), *Cylindrocolea* and *Cephaloziella* (both Cephaloziellaceae) had the highest diversification rates (0.48, 0.36 and 0.69, respectively).

### Distribution of mean diversification rates and mean age per elevational band

**Fig. 2:**
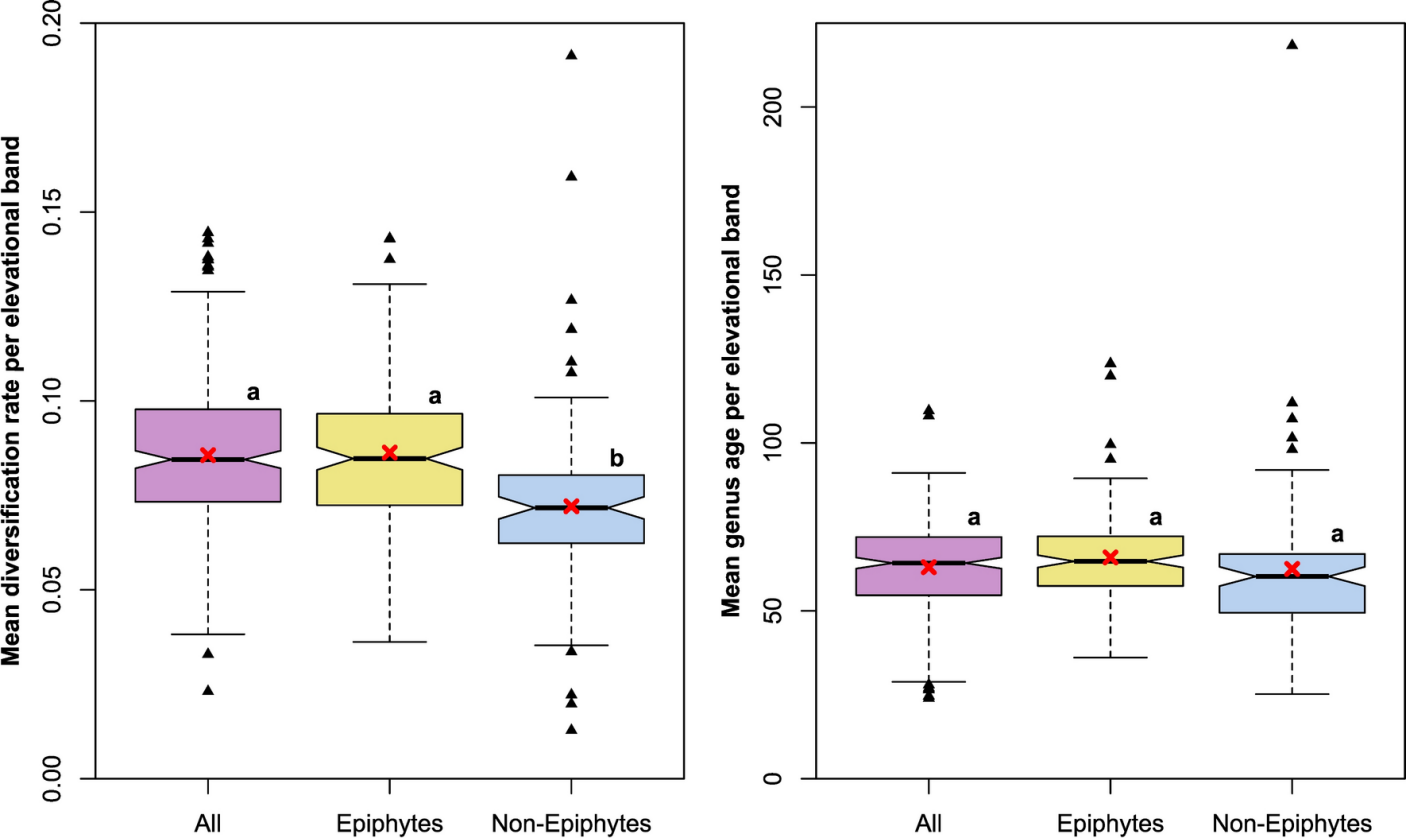
Boxplots showing the distribution of mean genus diversification rates (DivElev) and mean genus ages per elevational band (AgeElev) in all, epiphytic, and non-epiphytic liverworts of our data set. Small letters above boxes indicate results of Tukey’s Honest Significant Differences, red crosses correspond to the group means, triangles depict outliers.

The one-way ANOVA analysis revealed a significant difference in mean diversification rates of genera per elevational band (DivElev) between all, epiphytic, and non-epiphytic genera (F_(2, 529)_ = 16.12, P < 0.001). Tukey’s HSD Test for multiple comparisons indicated that the mean value of non-epiphytic DivElev significantly differed from the mean values of epiphytic and overall DivElev (P < 0.001). The median of the mean diversification rates of genera per elevational band (DivElev) from the non-epiphytic dataset was lower than the median of the epiphytic and the overall dataset, with the 95% confidence intervals not overlapping (Fig. 2). The absolute range of values (including outliers) was largest in the non-epiphytic dataset (0.179), lowest for epiphytes (0.107), an intermediate for all (0.121), whereas the interquartile range was 0.024 in both, the epiphytic and overall dataset, compared to the non-epiphytic dataset (0.018).

The mean genus ages per elevational band (AgeElev) did not differ significantly between the three groups (F_(2,529)_ = 2.023, P > 0.05), with the 95% confidence intervals of the medians overlapping (Fig. 2).

### Regression analyses

**Fig. 3:**
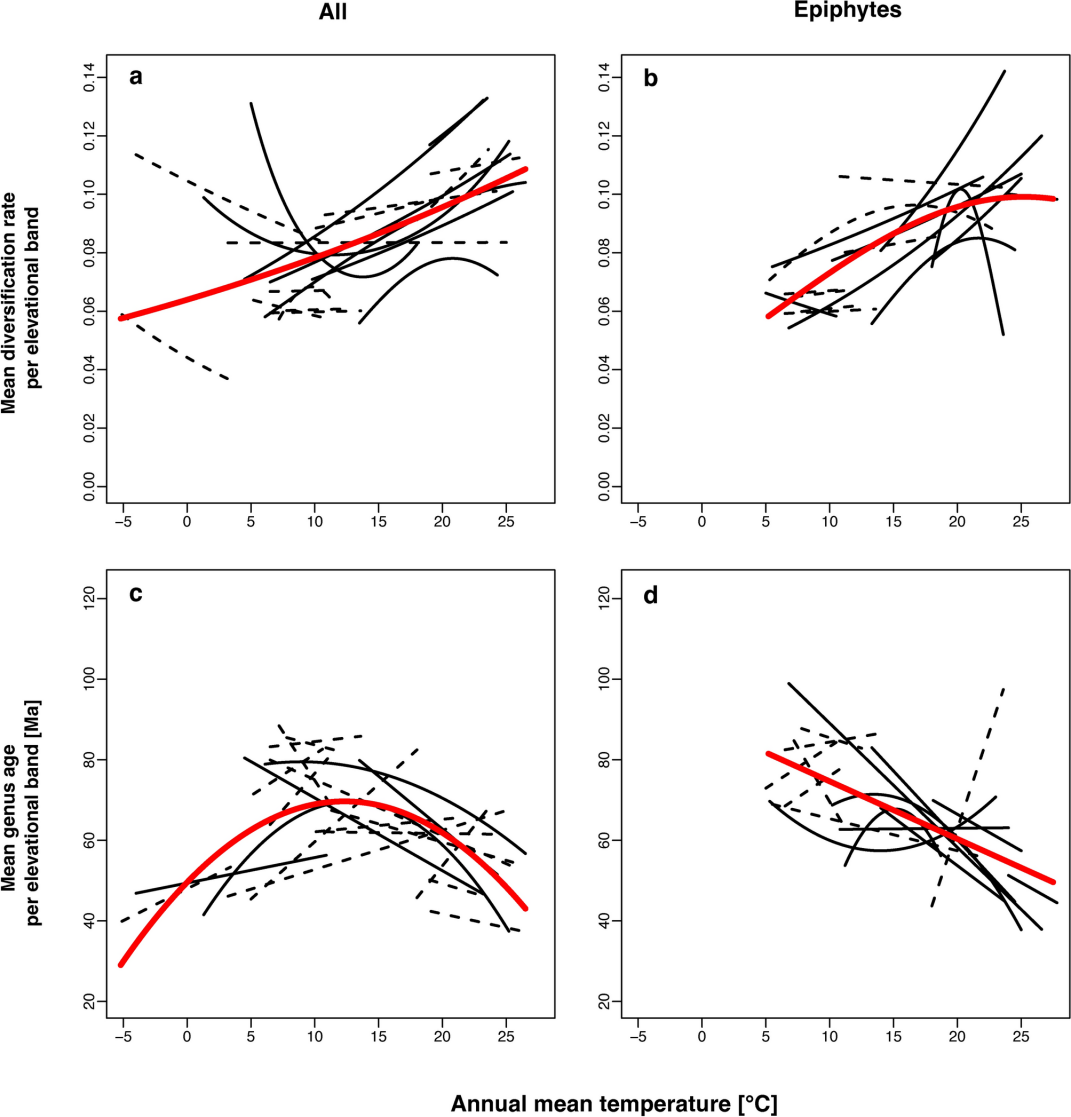
Temperature relationships of overall and epiphytic liverwort mean genus diversification rates per elevational band (DivElev) and mean genus ages per elevational band (AgeElev). Thin solid lines indicate significant (P < 0.01), dashed lines non-significant (P > 0.01) regression trend lines from individual locations; red lines depict the global trends.

On a global scale, Bio1, Bio5, and Bio6 were significant predictors for DivElev of all and epiphytic genera. DivElev of all genera increased linearly with increasing mean annual temperature (Bio1, ${\text{R}}_{\text{p}}^{2}$=0.28, P < 0.001; Fig. 3a, Table 2) and increasing minimum temperature (Bio6, ${\text{R}}_{\text{p}}^{2}$=0.33, P < 0.001). Maximum temperature of the warmest month (Bio5) explained 20% of the variance (P < 0.001) and had a U-shaped relationship with DivElev. At the regional level, four of 20 locations also showed a linear increasing relationship between DivElev and temperature (${\text{R}}_{\text{p}}^{2}$=0.44–0.86, P < 0.01), whereas a hump-shaped curve explained best the data of Mt. Roraima (Guyana) and Urewera NP, New Zealand (${\text{R}}_{\text{p}}^{2}$=0.72, P < 0.001 and ${\text{R}}_{\text{p}}^{2}$=0.63, P < 0.05, respectively). Switzerland and Mt. Ruapehu (New Zealand) showed a linearly decreasing relationship between DivElev and temperature (${\text{R}}_{\text{p}}^{2}$=0.37 and 0.5, P < 0.05), Napo (Ecuador) and Pico Island (Azores, Portugal) showed a slightly U-shaped pattern (${\text{R}}_{\text{p}}^{2}$= 0.56 and 0.70, P < 0.001, respectively). Temperature was not a significant predictor on ten locations.

**Table 2:** Summary of models regressing overall and epiphytic DivElev (mean diversification rate per elevational band) and AgeElev (mean genus age per elevational band).

Responding variable	Coefficients	Coefficient estimate	Standard error	P value	${\text{R}}_{\text{p}}^{2}$/ (adj.) R^2^
DivElev (all)	Intercept	-2.684	0.034	< 0.001	
	Bio1	0.022	0.002	< 0.001	0.28
DivElev (all)	Intercept	-1.916	0.150	< 0.001	
	Bio5	-0.073	0.016	< 0.001	
	Bio5^2^	0.002	0.421 × 10^–3^	< 0.001	0.20
DivElev (all)	Intercept	-2.534	0.021	< 0.001	
	Bio6	0.018	0.002	< 0.001	0.33
AgeElev (all)	Intercept	49.575	2.181	< 0.001	
	Bio1	3.262	0.345	< 0.001	
	Bio1^2^	-0.132	0.013	< 0.001	0.27
AgeElev (all)	Intercept	15.796	7.967	< 0.05	
	Bio5	5.917	0.865	< 0.001	
	Bio5^2^	-0.166	0.022	< 0.001	0.18
AgeElev (all)	Intercept	67.38	1.175	< 0.001	
	Bio6	0.532	0.1	< 0.001	
	Bio6^2^	-0.057	0.007	< 0.001	0.21
DivElev (Epiphytes)	Intercept	-3.122	0.14	< 0.001	
	Bio1	0.073	0.018	< 0.001	
	Bio1^2^	-0.001	0.557 × 10^–3^	< 0.01	0.37
DivElev (Epiphytes)	Intercept	-2.992	0.08	< 0.001	
	Bio5	0.029	0.004	< 0.001	0.3
DivElev (Epiphytes)	Intercept	-2.572	0.033	< 0.001	
	Bio6	0.019	0.002	< 0.001	0.29
AgeElev (Epiphytes)	Intercept	88.939	2.884	< 0.001	
	Bio1	-1.43	0.17	< 0.001	0.31
AgeElev (Epiphytes)	Intercept	100.86	4.511	< 0.001	
	Bio5	-1.635	0.206	< 0.001	0.28
AgeElev (Epiphytes)	Intercept	76.92	1.817	< 0.001	
	Bio6	-1.012	0.141	< 0.001	0.24

Bio1, annual mean temperature (°C); Bio5, max temperature of warmest month (°C); Bio6, min temperature of coldest month (°C).

DivElev of epiphytic species increased with each of the three temperature variables (Bio1 + Bio1^2^ ${\text{R}}_{\text{p}}^{2}$=0.37, P_Bio1_ < 0.001, P_Bio1_^2^ < 0.01, Bio5 ${\text{R}}_{\text{p}}^{2}$=0.30, P < 0.001, and Bio6 ${\text{R}}_{\text{p}}^{2}$=0.29, P < 0.001; Fig. 2b, Table 2). The analyses with the dataset reduced to one record per genus per elevational belt showed similar results: The ${\text{R}}_{\text{p}}^{2}$-values of DivElev of all species regressed against Bio1, Bio5, and Bio6 were 25%, 19% and 32% (all P < 0.001), respectively. The temperature variables explained 37% (Bio1, P < 0.001), 31% (Bio5, P < 0.001), and 32% (Bio6, P < 0.001) of the variance in DivElev of the reduced epiphytic dataset. The orientation of the global regression lines was the same for both datasets, although some of the local trend lines changed from linear to hump-shaped or vice versa.

Elev^2^ explained 6% of the variance in epiphytic DivElev (P < 0.01) with a hump-shaped regression line, whereas the relationship to all DivElev was not significant. We did not detect noteworthy effects (i.e. ${\text{R}}_{\text{p}}^{2}$>0.05, and P < 0.05) of relRich, or relElev on either all or epiphytic DivElev.

Mean genus age (AgeElev) of all species showed a significant (P < 0.001) hump-shaped relationship with temperature peaking at 12.3 °C (Bio1), 17.8 °C (Bio5), and 4.7 °C (Bio6). Adjusted R^2^ values were 0.27, 0.18, and 0.21, respectively (Fig. 2c, Table 2). Napo (Ecuador) was the only regional location with a unimodal relationship between AgeElev and temperature (adj. R^2^ = 0.63, P < 0.001), for 13 locations we did not observe a significant pattern. Mean genus age decreased with increasing temperature in four locations: Huon Peninsula (adj. R^2^ = 0.86, P < 0.05) and Mount Wilhelm (R^2^ = 0.59 P < 0.05), both in Papua New Guinea; Mt. Roraima, Guyana (R^2^ = 0.89, P < 0.001); and the location of the Bryotrop III Expedition to DRC and Rwanda (R^2^ = 0.21 P < 0.01), whereas the opposite pattern applied to Switzerland (R^2^ = 0.79 P < 0.01) and Pico Island, Portugal (R^2^ = 0.40 P < 0.05). In the models of the reduced dataset of the overall mean genus age, the variance explained by Bio1, Bio5 and Bio6 decreased to 0.14, 0.16 (both P < 0.001) and 0.10 (P < 0.1), respectively.

AgeElev of the epiphytes significantly decreased (P < 0.001) with increasing temperature, R^2^ values were 0.31 (Bio1), 0.28 (Bio5), and 0.24 (Bio6; Fig. 2d, Table 2). The same pattern was observed to apply to six regional locations, whereas eight locations did not show a significant pattern. Two transects showed a unimodal pattern, of which the curve of Mt. Gede (Indonesia) was hump-shaped (adj. R^2^ = 0.86, P < 0.01), in contrast to a U-shaped curve explaining best the AgeElev data of the transect in the Colombian Andes (adj. R^2^ = 0.45, P < 0.01).

The overall trend lines shown in Fig. 3 show the general pattern from which some individual transects differ. Partly, these different trends are not statistically significant (dashed lines) and thus do not warrant further exploration, but some are well-supported and presumably reflect individual variation between transects. These variations might have biogeographical reasons or may also be caused by sampling and observer biases; a full exploration of this variation requires more detailed data than currently available.

The variance of the reduced epiphytic dataset explained by the temperature predictors was similar and resulted in R^2^ values of 0.31, 0.27, and 0.26 (all P < 0.001). We found no significant relationship of overall or epiphytic relRich R^2^ > 0.05, P < 0.05 with AgeElev. There were no noteworthy effects of the remaining precipitation-related variables on DivElev or AgeElev of the epiphytic genera detectable.

Precipitation had a positive linear relationship with DivElev of all genera (${\text{R}}_{\text{p}}^{2}$=0.18, P < 0.001 (Bio12); ${\text{R}}_{\text{p}}^{2}$=0.21, P < 0.001 (Bio13); ${\text{R}}_{\text{p}}^{2}$=0.11, P < 0.001 (Bio18);${\text{R}}_{\text{p}}^{2}$=0.9, P < 0.001 (Bio19)), and a hump-shaped relationship with AgeElev (adj. R^2^ = 0.16, P < 0.001 (Bio12); adj. R^2^ = 0.15, P < 0.001 (Bio14); adj. R^2^ = 0.24, P < 0.001 (Bio19)). The results for DivElev ~ Bio14, and AgeElev ~ Bio13/Bio18 were below (${\text{R}}_{\text{p}}^{2}$ or R^2^ < 0.05). Precipitation of the warmest month (Bio18) had a U-shaped relationship with epiphytic DivElev (${\text{R}}_{\text{p}}^{2}$=0.07, P < 0.01).

The flatly hump-shaped curve of non-epiphytic diversification rates (DivElev) peaked at mid-Elev and decelerated towards both ends of the gradient but increased slightly with relElev (${\text{R}}_{\text{p}}^{2}$=0.10, ${\text{R}}_{\text{p}}^{2}$=0.05, respectively, both P < 0.05). In contrast, DivElev of the Nilgiri Hills (India) linearly increased with elevation (${\text{R}}_{\text{p}}^{2}$=0.69, P < 0.001), whereas the relationship between DivElev and elevation of Mt. Roraima (Guyana) and Haast Pass (New Zealand) were best explained by a hump-shaped curve (${\text{R}}_{\text{p}}^{2}$=0.74, and ${\text{R}}_{\text{p}}^{2}$=0.78, respectively, both P < 0.001). We did not detect a significant elevational pattern of DivElev for the remaining locations. Non-epiphytic genus age per elevational band (AgeElev) was flatly U-shaped in relation to Elev, and decreased linearly with relElev (adj. R^2^ = 0.10, P < 0.05 and R^2^ = 0.10, P < 0.01, respectively). AgeElev was decreasing with elevation in Nilgiri Hills (R^2^ = 0.36, P < 0.05), and Haast Pass (R^2^ = 0.81, P < 0.05). We found a hump-shaped pattern on the transect on Mt. Roraima (adj. R^2^ = 0.45, P < 0.05), whereas the opposite pattern (U-shaped) applied to Gongga Mountains (adj. R^2^ = 0.51, P < 0.05). The results of regression analyses of data set with and without younger volcanic islands did not show noteworthy differences (Supplementary Table S3). Further, there was no significant global pattern detectable in the relationship between DivElev or AgeElev of non-epiphytes and relRich, or one of the temperature-related variables.

The values of DivElev and AgeElev in relation to Rich were too heteroscedastic to be analyzed via regressions.

### Ancestral state reconstruction

**Fig. 4:**
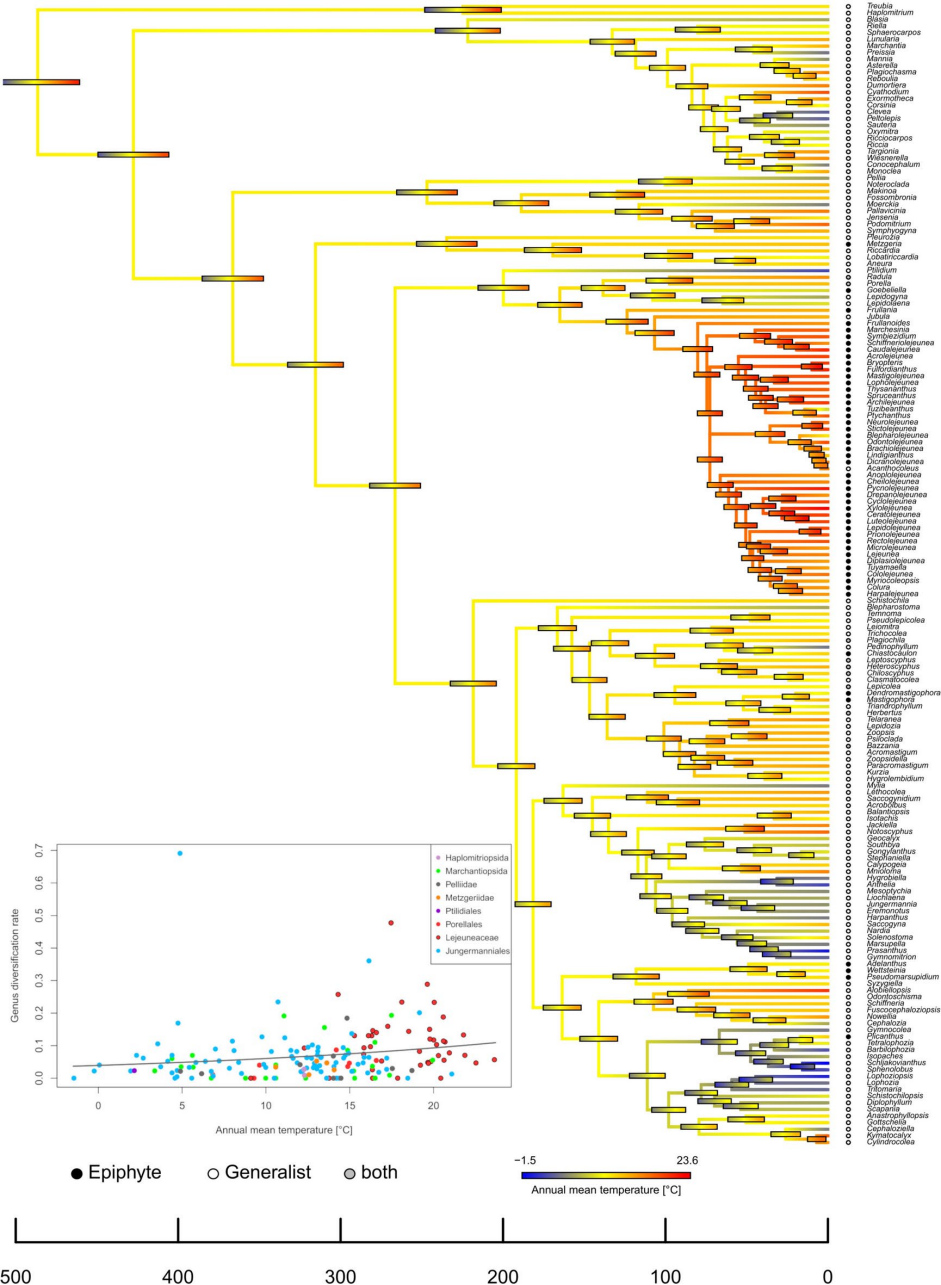
Ancestral character estimation of temperature preferences (Bio1) in liverworts. The inset illustrates the thermal distribution of the genera’s diversification rates. Habitat preferences of genera are based on Feldberg et al. ^52^ and further literature.

The estimation of Pagel’s lambda was 0.82, P < 0.001 Bio1 and Bio6, indicating significant phylogenetic signal in temperature preferences. Unsurprisingly, the error bars at the deepest nodes cover a broad temperature amplitude around a mean of about 11 °C (Fig. 4), precluding a reliable estimation of ancestral temperature preferences. Clear preferences of genera for colder and warmer climates were evident only later on and each evolved several times independently, but the preference for higher temperatures clusters in the clade of the Lejeuneaceae. The comparison of temperature and habitat preference shows that the majority of epiphytic lineages occur in warmer climates, whereas most genera inhabiting colder climate are generalists.

## Discussion

Mountains, especially in the tropics, are the global centers of plant diversity in general and liverwort diversity in particular^54,106^, due to their topographical complexity, diversity of habitats, often relatively young age, and dynamic history^24,29,38^. However, many questions remain open on how this diversity has evolved. In the present study we set out to assess the elevational distribution of diversification rates and genus ages as well as the evolution of climatic preferences of liverworts to contribute a novel aspect to our understanding of how liverwort diversity has evolved. We found that, overall, diversification rates in liverworts were positively related to temperature and hence decreased with elevation, and that genus ages were highest at mid-elevation where liverwort species richness also peaks.

The decreasing diversification rates with elevation found in our study for liverworts is consistent with the positive diversification rate-temperature relationship found in the liverwort and overall bryophyte floras of China by X. Song et al.^57^, who used mean species numbers per genus as estimation for recent diversification rates in bryophytes. The increase in diversification rates towards high temperatures is also in agreement with the significantly higher diversification rates of tropical versus temperate liverworts genera found by Laenen et al.^42^. On the other hand, mosses show decreasing diversification rates with increasing temperature in China^57^ and among birds, diversification rates increase with elevation on a worldwide basis^9^. These contrasting results suggest that the diversity of different groups of organisms evolves idiosyncratically on mountains. However, all these studies used somewhat different methods to estimate diversification rates, so that comparisons should be made with care. For example, Quintero and Jetz^9^ estimated diversification rates at species-level, whereas we averaged rates to genus level. Unfortunately, there are still too few studies to develop any generalizations, but our study on liverworts reveals a diversification history that clearly differs from that of other organism groups studied so far.

Our study suggests that the extant liverwort genera probably descend from clades which evolved under rather cool conditions. This climatic environment is today prevalent at mid-elevations of contemporary (tropical) mountains where we also find the highest species richness of liverworts^54,107–109^ and the highest mean genus ages. All of this is consistent with an interpretation of cool and humid environmental conditions corresponding to the ancestral niche conditions of most of the extant liverwort lineages. Hence, as already proposed by Laenen et al.^42^, a tropical origin in the sense of high temperatures which has been found in angiosperm lineages ^15^, does thus not apply to liverworts. This assumption, which is also is in line with the findings of Qian and Kessler^59^, corresponds to the physiological features of most liverworts, since they are unable to actively regulate their cell water potential and thus to maintain their metabolism under water-deficient conditions^110,111^ associated with high temperatures and high evapotranspiration. During earth’s history, the ancient climatic niche of liverworts on mountains was not static but probably shifted to higher and lower elevations multiple times due to climate fluctuations and orogenic processes^112^. The montane zone that today is found at intermediate elevations can thus also be regarded as a refugium for liverworts in which species were able to survive climatic changes^113^. In accordance with the niche conservatism hypothesis, it would therefore appear that both cold (high elevation and high latitudes) and hot (tropical lowland) habitats have been colonized from cool and humid regions by different liverwort lineages and thus, comparatively recently. Both extremes of the temperature gradient hence probably represent the current frontiers of liverwort diversification. Interestingly, however, we only found that mean genus diversification rates are high at high temperatures in the (tropical) lowlands, and low at low temperatures at high elevations.

The liverwort flora of high elevations is dominated by non-epiphytes, which have on average significantly lower diversification rates than epiphytes (Fig. 2). However, we also observed the opposite in a few genera (from generalistic Jungermanniales, inset Fig. 4, Supplementary Table S1) which, occurring at low temperatures and thus high elevations at rather high latitudes, exhibit accelerated diversification rates, and hence seem to diversify into the niches provided by the alpine environment. These exceptions are in line with accelerated rates of diversification at high altitude ecosystem in angiosperm lineages of the Andean páramo ^114^, the Hengduan Mountains in China^115^, or western North America^116^, as well as in a neotropical lycophyte clade^117^. Physiological adaptations which evolved in several liverwort species enable them to cope with the harsh conditions of alpine habitats: they are able to withstand low temperatures, frost, and high levels of UV-B radiation^118,119^. Despite these adaptations, the on average low diversification rates at low temperatures found by us point either to rare speciation or high extinction rates or both in combination, e.g. due to the lack of epiphytic substrate or low habitat heterogeneity, which both limit vascular plant species richness^120,121^, or the very challenging abiotic conditions at high elevations.

In contrast, we found increased diversification in epiphytic lineages towards the warm (tropical) lowland end of the elevational gradient, where epiphytic genera are also youngest. Several liverwort lineages have evolved adaptations enabling them to recover from complete desiccation and to fully resume their metabolism when rehydrated^110,122^. In epiphytes, the gradient of desiccation tolerance roughly follows the humidity gradient along the tree, with epiphytes of branches and in the canopy being more tolerant than those of the trunk and these in turn more than those on the tree base, reflecting the intensity of drought stress in the respective position^123,124^.

The high diversification rates in Lejeuneaceae indicates that this family drives the elevational distribution pattern of diversification. We found that Lejeuneaceae genera show a preference for low elevations and high temperatures (inset Fig. 4, Supplementary Table S1). This corresponds to earlier studies, which found that Lejeuneaceae, as one of the most species rich and predominantly epiphytic liverwort families, have a tropical center of species richness, accounting for up to 70% of the liverwort diversity in the lowland and about 45% of lower montane rainforests of the Neotropics^30,125^. Our study further indicates that the Lejeuneaceae family diversified following a niche expansion towards warmer climates and thus probably shifted its range of occurrence towards lower elevations (Fig. 4). Wilson et al.^55^ found a sudden increase of lineage accumulation in Lejeuneaceae during late Cretaceous intermitting an otherwise rather constantly proceeding diversification process over time. These findings were supported by Feldberg et al.^52^, who in addition found evidence for a correlation between accelerated speciation rates in the order Porellales, to which the Lejeuneaceae belong, and epiphytism, which they explain by the increased availability of epiphytic niches in moist forests with continuously high temperatures after the rise of the flowering plants to ecological dominance during the late Cretaceous. Further, Silva et al.^56^ found that the epiphytic genus *Frullania* (in the family Frullaniaceae, also part of the Porellales) rapidly diversified during the Palaeogene and Neogene. Our study indeed confirms accelerated net diversification rates in several Lejeuneaceae genera, but we did not find evidence for higher rates within the epiphytic Porellales other than Lejeuneaceae, nor for epiphytic genera of other orders, e.g. in the genus *Metzgeria* (Metzgeriales), in which the epiphytic lifestyle has probably evolved under similar conditions as in Lejeuneaceae^126^. Furthermore, we found higher diversification rates in terrestrial and generalistic genera nested within Marchantiales, Pelliidae, and Jungermanniales (inset Fig. 4, Supplementary Table S1). The majority of these genera tends to occur at low to mid-elevations, and cool to warm climates, too. Hence, we conclude that epiphytism is probably one important of several factors associated with rapid diversification in Lejeuneaceae.

Turning towards the non-epiphytic species, we found that diversification rates and genus ages do not correlate with temperature. The elevational distribution of non-epiphytic liverwort species richness is more strongly affected by microhabitat parameters rather than by regional climate ^72^. The same may be true for the distribution of diversification rates and genus ages among non-epiphytic liverworts, masking clear climatic patterns. We found elevation to have a weak, but significant relationship with both diversification rates and ages. However, elevation cannot be seen as a determinant as such, but rather as an aggregation of diverse covarying variables^127^. Furthermore, although some of the local gradients showed significant and well supported thermal and elevational patterns (Supplementary Fig. S2), there is no clear global trend detectable. The lower number of gradients and records of non-epiphytic species (especially towards the ends of the elevational gradient) included in our study may weaken the power for statistical inference.

In summary, we suggest that the extant liverwort lineages have originated at cool (and humid) conditions, which are today found at mid-elevations of the world’s mountains. In this climate, liverwort species richness has accumulated due to suitable environmental conditions, long time, and moderate diversification rates. Subsequent diversification predominantly took place towards the warm lowlands, most notably among epiphytic genera, especially from the large Lejeuneaceae family. In contrast, the colder end of the global temperature gradient is poor in epiphytic liverworts and has on average low diversification rates mainly from relatively young lineages. In contrast to angiosperms and lycophytes, we did not find a general trend towards accelerated diversification processes taking place among the young liverwort flora at high alpine regions.

## Conclusions

This is the first global comparison of the elevational diversification dynamics in liverworts. We found that diversification rates decrease linearly with decreasing temperature and thus increasing elevation, whereas mean age peaks at moderate temperature at mid-elevations and decreases towards both ends of the gradient. We interpret our results to show that niche conservatism and time-for-speciation have played the dominant role in shaping the gradient of liverwort diversity. We found no correlation of diversification rates to species richness, showing that diversification rates only play a minor role in determining diversity patterns, as also recently found for flowering plants on a global scale^10^. The current hotspot of liverwort diversification corresponds to the epiphytic tropical lowland taxa, and although overall liverwort assemblages in the lowlands are relatively species poor, this is where liverwort evolution appears to be most active. This mainly accounts for members of the Lejeuneaceae family and might be either indicative of temperature accelerating speciation rates or a burst of adaptive radiation exploiting novel epiphytic niches. It has been suggested that the past niche diversification history can provide important insights into how lineages may respond evolutionarily to climate change^128^. In the case of liverworts, the ongoing radiation process towards warmer and dryer niches in the tropical lowlands reveals an adaptability to such conditions and provides cause for hope. However, this primarily applies to only a few liverwort lineages, whereas most liverwort genera favor their ancient habitats of cool and moist climates. This climatic niche conservatism together with the low evolutionary activity towards the colder end of the elevational gradient and of non-epiphytic liverworts in general suggests that the species richness of montane liverwort genera likely will be threatened by fast climate warming.

## Supplementary Information

Supplementary Information.

## Data Availability

The data sets of this study (i.e., species records per elevation of transect, DivElev and AgeElev) are available from the dryad repository (10.5061/dryad.t76hdr85j) or are included in the Supplementary information of this article.
