# Supplementary material for: Temperature dependence of liverwort diversification reveals a cool origin and hot hotspots
Source: Sci Rep. 2025 Jan 25;15:3225. doi: 10.1038/s41598-025-87206-1 (PMC11762728; doi:10.1038/s41598-025-87206-1)
Supplement: Supplementary file 1 — Supplementary Information. [file 41598_2025_87206_MOESM1_ESM.pdf]

Scientific Reports **Collection Bryophyte resilience to environmental factors**

Temperature-dependence of liverwort diversification: cool origin and hot hotspots

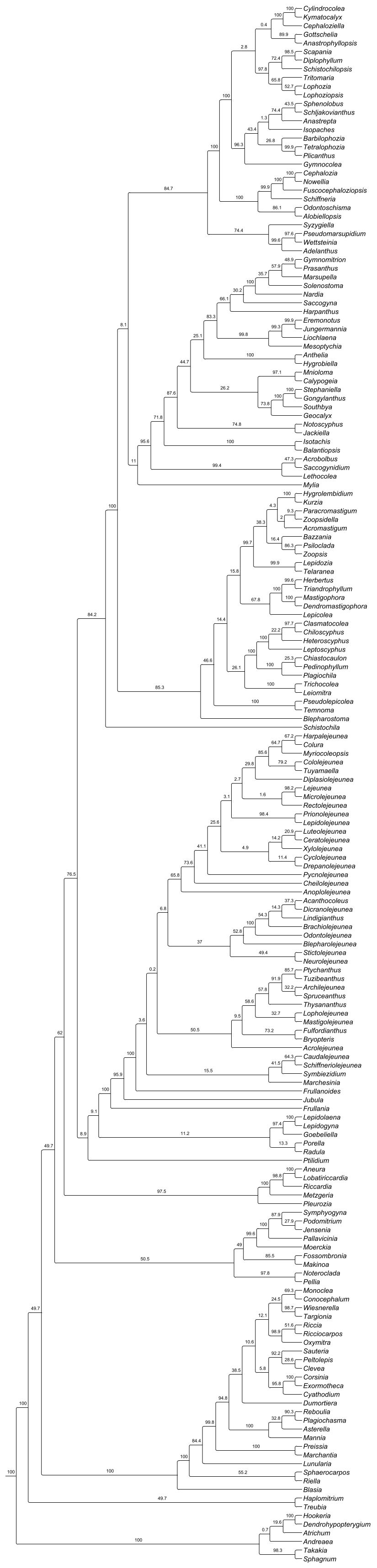

Figure S1. Phylogeny used in this study (based on [47]); bootstrap support values are shown above the branches.

Figure S2. Non-epiphytic liverwort mean genus diversification rates per elevational band (DivElev) and mean genus ages per elevational band (AgeElev) in relation to elevation, relative elevation and annual mean temperature (Bio1).

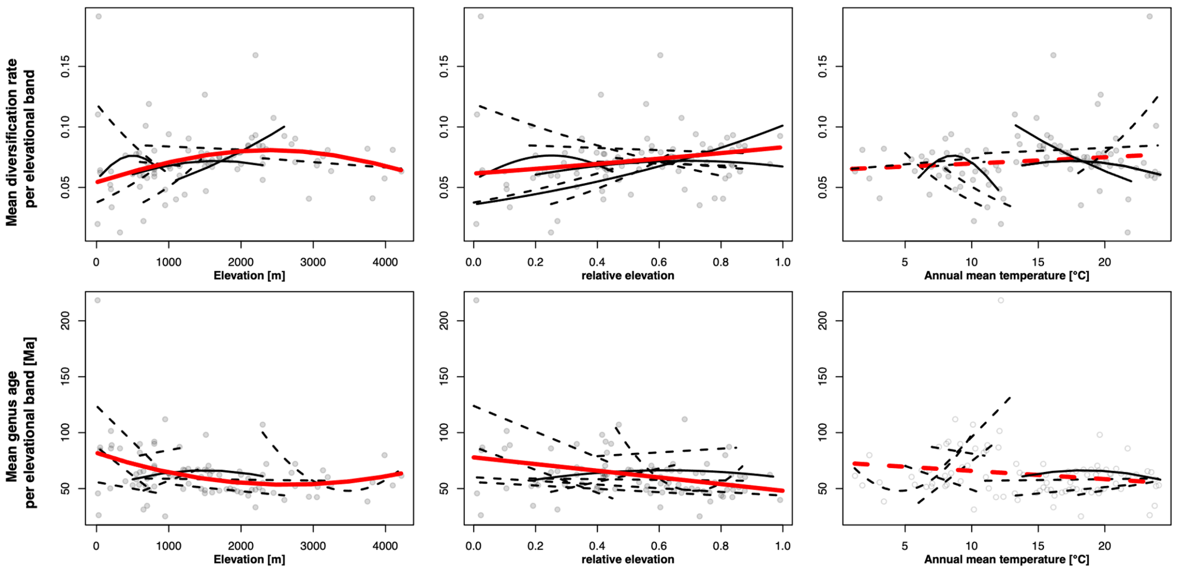

Appendix S1. Additional references of source data Koponen Norris-Expedition (3) and Bryotrop III Expedition (5).

(3) Piippo, S. 1984. Bryophyte flora of the Huon Peninsula, Papua New Guinea. III. Haplomitriaceae, Lepicoleaceae, Herbertaceae, Pseudolepicoleaceae, Trichocoleaceae, Schistochilaceae, Balantiopsaceae, Pleuroziaceae and Porellaceae (Hepaticae) Bot. Fennici 21: 21-48.

Piippo, S. 1984. Bryophyte flora of the Huon Peninsula, Papua New Guinea. VI. Lepidoziaceae subfam. Lepidozioideae, Calypogeiaceae, Adelanthaceae, Caphaloziaceae subfam. Cephalozioideae and subfam. Odontoschismatoideae and Jubulaceae (Hepaticae). Ann. Bot. Fennici 21: 309-335.

Piippo, S. 1985. Bryophyte flora of the Huon Peninsula, Papua New Guinea. X. Jackiellaceae, Scapaniaceae, Arnelliaceae and Acrobolbaceae (Hepaticae). Acta Bot. Fennica 131: 89-97.

Piippo, S. 1985. Bryophyte flora of the Huon Peninsula, Papua New Guinea. XII. Geocalycaceae (Hepaticae). Acta Bot. Fennica 131: 129-167.

Piippo, S. 1985. Bryophyte flora of the Huon Peninsula, Papua New Guinea. XIII. *Arachniopsis* and *Kurzia* (Lepidoziaceae subfam. Lepidozioideae, Hepaticae). Acta Bot. Fennica 131: 169-179.

Hattori, S. and Piippo, S. 1986. Bryophyte flora of the Huon Peninsula, Papua New Guinea. XV. *Frullania* (Frullaniaceae, Hepaticae). Acta Bot. Fennica 133: 25-58.

Grolle, R. and Piippo, S. 1986. Bryophyte flora of the Huon Peninsula, Papua New Guinea. XVI. Pallaviciniaceae (Hepaticae). Acta Bot. Fennica 133: 59-79.

Piippo, S. 1988. Bryophyte flora of the Huon Peninsula, Papua New Guinea. XXI. *Lepicolea norrisii* (Lepicoleaceae, Hepaticae). Ann. Bot. Fennici 25: 55-57.

Piippo, S. 1988. Bryophyte flora of the Huon Peninsula, Papua New Guinea. XXII. Targioniaceae, Wiesnerellaceae, Aytoniaceae and Ricciaceae (Marchantiales, Hepaticae). Ann. Bot. Fennici 25: 97-107.
Piippo, S. 1988. Bryophyte flora of the Huon Peninsula, Papua New Guinea. XXIII. Treubiaceae, Allisoniaceae and Makinoaceae (Metzgeriales, Hepaticae). Ann. Bot. Fennici 25: 159-164.
Piippo, S. and Váňa, J. 1989. Bryophyte flora of the Huon Peninsula, Papua New Guinea. XXIX. Jungermanniaceae and Gymnomitriaceae (Hepaticae). Ann. Bot. Fennici 26: 107-125.
Piippo, S. 1989. Bryophyte flora of the Huon Peninsula, Papua New Guinea. XXX. Plagiochilaceae (Hepaticae). Ann. Bot. Fennici 26: 183-236.
Váňa, J. and Piippo, S. 1989. Bryophyte flora of the Huon Peninsula, Papua New Guinea. XXXI. Cephaloziaceae subfam. Alobielloideae, Cephaloziellaceae, Antheliaceae and Lophoziaceae (Hepaticae). Ann. Bot. Fennici 26: 263-290.
Yamada, K. and Piippo, S. 1989. Bryophyte flora of the Huon Peninsula, Papua New Guinea. XXXII. Radula (Radulaceae, Hepaticae). Ann. Bot. Fennici 26: 349-387.
Grolle, R. and Piippo, S. 1990. Bryophyte flora of the Huon Peninsula, Papua New Guinea. XXXVII. *Leucolejeunea* (Lejeuneaceae, Hepaticae). Ann. Bot. Fennici 27: 119-129.
Piippo, S. 1991. Bryophyte flora of the Huon Peninsula, Papua New Guinea. XXXIX. *Fossombronia* (Fossombroniaceae), and *Metzgeria* (Metzgeriaceae, Hepaticae). Acta Bot. Fennica 143: 1-22.
Piippo, S. 1991. Bryophyte flora of the Huon Peninsula, Papua New Guinea. XLIX. *Targionia* (Targioniaceae, Hepaticae). Ann. Bot. Fennici 28: 273-276.
Bischler, H. and Piippo, S. 1991. Bryophyte flora of the Huon Peninsula, Papua New Guinea. L. *Marchantia* (Marchanticeae, Hepaticae). Ann. Bot. Fennici 28: 277-301.
Piippo, S. 1992. Bryophyte flora of the Huon Peninsula, Papua New Guinea. LI. Additions and corrections to the Geocalycaceae (Hepaticae). Ann. Bot. Fennici 29: 243-248.
Pócs, T., Piippo, S., and Mizutani, M. 1995. Bryophyte flora of the Huon Peninsula, Papua New Guinea. LVI. Preliminary contributions on Lejeuneaceae (Hepaticae) 2. Ann. Bot. Fennici 32: 259-268.
Pócs, T. and Piippo, S. 1999. Bryophyte flora of the Huon Peninsula, Papua New Guinea. LXIV. *Aphanolejeunea* (Lejeuneaceae, Hepaticae). Acta Bot. Fennica 165: 85-102.
Pócs, T., Mizutani, M., and Piippo, S. 1994. Bryophyte flora of the Huon Peninsula, Papua New Guinea. LXV. Preliminary contributions on Lejeuneaceae (Hepaticae) 1. Ann. Bot. Fennici 31: 179-190.
Gradstein, R., He, X.-L., Piippo, S., and Mizutani, M. 1999. Bryophyte flora of the Huon Peninsula, Papua New Guinea. LXVII. Lejeuneaceae subfamily Ptychanthoideae (Hepaticae). Acta Bot. Fennica 174: 1-88.
Pócs, T. and Piippo, S. 2011. Bryophyte flora of the Huon Peninsula, Papua New Guinea. LXXIV. *Cololejeunea* (Lejeuneaceae, Hepaticae). Acta Bryolichenologica Asiatica 4: 59-137.

(5) Fischer, E. 1993. Taxonomic results of the BRYOTROP-Expedition to Zaire and Rwanda 10. Trichocoleaceae, Geocalycaceae, Acrobolbaceae, Balantiopsidaceae, Lepidoziaceae (*Telaranea*, *Arachniopsis*), Calypogeiaceae, Adelanthaceae, Porellaceae, Jubulaceae, Marchantiaceae (*Dumortiera*), Polytrichaceae. Trop. Bryol. 8: 83-97.
Bischler-Causse, H. 1993. Taxonomic results of the BRYOTROP-Expedition to Zaire and Rwanda 6. Aytoniaceae, Marchantiaceae. Trop. Bryol. 8: 53-54.
Grolle, R. 1993. Taxonomic results of the BRYOTROP-Expedition to Zaire and Rwanda 9. Pallaviciniaceae, Haplomitriaceae. Trop. Bryol. 8: 75-82.
Pócs, T. 1994. Taxonomic results of the BRYOTROP-Expedition to Zaire and Rwanda 28. Lejeuneaceae, a ramicolous collection. Trop. Bryol. 9: 131-136.

Pócs, T. 1994. Taxonomic results of the BRYOTROP-Expedition to Zaire and Rwanda 27. Lepidoziaceae, II. Trop. Bryol. 9: 123-130.
Pócs, T. 1993. Taxonomic results of the BRYOTROP-Expedition to Zaire and Rwanda 12. Metzgeriaceae, Plagiochilaceae, Lejeuneaceae (the non-epiphyllous collections). Trop. Bryol. 8: 105-125.
Tixier, P. 1995. Résultats taxonomiques de l´ éxpédition BRYOTROP au Zaire et Rwanda 30. Bryophytes épiphylles (récoltes de E. Fischer). Trop. Bryol. 11: 11-76.
Váňa, J. 1993. Taxonomic results of the BRYOTROP-Expedition to Zaire and Rwanda 11. Cephaloziaceae, Cephaloziellaceae, Gymnomitriaceae, Jungermanniaceae, Lophoziaceae. Trop. Bryol. 8:99-103.
Yamada, K. 1993. Taxonomic results of the BRYOTROP-Expedition to Zaire and Rwanda 13. Radulaceae. Trop. Bryol. 8: 127-130.

Table S1. Species per genus numbers, calculated genus diversification rates and mean Bio1 values of each genus analyzed in the study.

Genus	Species per genus	Calculated genus diversification rate	Mean Bio1 (°C)
Acanthocoleus	8	0.47738598	17.46
Acrobolbus	30	0.04591283	12.89
Acrolejeunea	21	0.05526021	20.62
Acromastigum	35	0.06393601	15.85
Adelanthus	10	0.04711558	11.86
Alobiellopsis	3	0.01279336	21.09
Anastrepta	1	0	4.40
Anastrophyllopsis	3	0.02181121	12.17
Aneura	20	0.05248377	13.02
Anoplolejeunea	1	0	16.36
Anthelia	2	0.02211143	1.93
Archilejeunea	6	0.07770818	20.94
Asterella	50	0.15565844	13.49
Balantiopsis	11	0.13708888	15.12
Barbilophozia	5	0.06163587	2.37
Bazzania	100	0.06186834	14.66
Blasia	1	0	6.73
Blepharolejeunea	5	0.09258145	12.29
Blepharostoma	3	0.00659592	6.85

Brachiolejeunea	6	0.25751967	14.30
Bryopteris	3	0.11139251	20.68
Calypogeia	30	0.06383236	13.29
Caudalejeunea	13	0.13283959	21.79
Cephalozia	25	0.08950411	6.98
Cephaloziella	40	0.69114275	4.87
Ceratolejeunea	37	0.18803073	20.58
Cheilolejeunea	125	0.05263136	16.90
Chiastocaulon	17	0.06323815	13.27
Chiloscyphus	2	0.02870269	13.02
Clasmatocolea	20	0.12405094	11.12
Clevea	2	0.02250241	3.36
Cololejeunea	300	0.23389355	18.06
Colura	75	0.13063242	16.31
Conocephalum	3	0.03515483	4.94
Corsinia	1	0	10.59
Cyathodium	12	0.05550652	19.95
Cyclolejeunea	7	0.06944782	20.29
Cylindrocolea	12	0.3606251	16.14
Dendromastigophora	1	0	10.43
Dicranolejeunea	1	0	17.86
Diplasiolejeunea	50	0.0932698	15.85
Diplophyllum	16	0.05166441	6.38
Drepanolejeunea	60	0.14612355	16.37
Dumortiera	2	0.0090554	17.43
Eremonotus	1	0	5.57
Exormotheca	7	0.11015276	16.50
Fossombronia	80	0.06801252	14.02
Frullania	300	0.04613935	14.97
Frullanoides	8	0.02594241	16.35
Fulfordianthus	2	0.07028085	21.87
Fuscocephaloziopsis	17	0.0479189	14.18

Geocalyx	3	0.01451402	8.54
Goebeliella	1	0	9.29
Gongylanthus	8	0.12707849	9.76
Gottschelia	2	0.02001445	12.09
Gymnocolea	3	0.01642769	4.40
Gymnomitrion	25	0.10558764	3.54
Haplomitrium	7	0.00866194	12.30
Harpalejeunea	15	0.13063242	16.10
Harpanthus	3	0.02355154	4.70
Herbertus	15	0.08270776	13.09
Heteroscyphus	65	0.07641515	14.93
Hygrobiella	1	0	4.57
Hygrolembidium	8	0.23437325	10.72
Isopaches	4	0.05309025	7.21
Isotachis	15	0.08196779	11.84
Jackiella	4	0.02715995	17.20
Jensenia	7	0.04147182	12.03
Jubula	5	0.0358766	14.68
Jungermannia	8	0.0482376	5.82
Kurzia	40	0.09501403	13.11
Kymatocalyx	4	0.20118765	19.16
Leiomitra	10	0.03218671	12.83
Lejeunea	200	0.15926188	17.27
Lepicolea	10	0.08540594	11.49
Lepidogyna	2	0.01071834	6.83
Lepidolaena	7	0.04002203	9.61
Lepidolejeunea	13	0.23307368	20.04
Lepidozia	75	0.07112272	13.07
Leptoscyphus	30	0.09511681	12.51
Lethocolea	6	0.01612473	16.07
Lindigianthus	1	0	13.76
Liochlaena	2	0.02093375	6.73

Lobatiriccardia	7	0.0340914	10.58
Lopholejeunea	35	0.15005291	19.83
Lophozia	10	0.05188028	4.14
Lophoziopsis	6	0.04037071	0.06
Lunularia	1	0	15.29
Luteolejeunea	1	0	20.43
Makinoa	1	0	14.03
Mannia	10	0.07010839	5.61
Marchantia	50	0.08564851	14.87
Marchesinia	6	0.0399692	19.21
Marsupella	20	0.16913853	4.75
Mastigolejeunea	30	0.10310957	20.11
Mastigophora	4	0.07022425	14.73
Mesoptychia	12	0.03320455	5.42
Metzgeria	75	0.02550274	14.07
Microlejeunea	25	0.09675604	16.23
Mnioloma	10	0.06156723	17.21
Moerckia	3	0.0212863	4.90
Monoclea	2	0.02218023	15.23
Mylia	2	0.0042507	4.69
Myriocoleopsis	5	0.13063242	15.28
Nardia	15	0.04850406	8.37
Neurolejeunea	4	0.14395383	18.52
Noteroclada	1	0	14.47
Notoscyphus	1	0	18.46
Nowellia	10	0.06402571	16.14
Odontolejeunea	3	0.11734924	20.01
Odontoschisma	21	0.03545353	14.06
Oxymitra	1	0	9.98
Pallavicinia	12	0.02969482	17.29
Paracromastigum	15	0.04732271	15.80
Pedinophyllum	4	0.07040415	4.40

Pellia	4	0.01385191	6.27
Peltolepis	2	0.02250241	3.36
Plagiochasma	16	0.19311892	17.49
Plagiochila	400	0.093981	14.60
Pleurozia	12	0.01059067	12.56
Plicanthus	3	0.06496084	13.30
Podomitrium	3	0.02341396	18.69
Porella	50	0.08686484	14.19
Prasanthus	2	0.02196377	-0.25
Preissia	2	0.06022923	4.40
Prionolejeunea	24	0.2887857	19.63
Pseudolepicolea	7	0.0668596	11.54
Pseudomarsupidium	4	0.06106246	12.72
Psiloclada	1	0	14.21
Ptilidium	3	0.02309414	2.00
Ptychanthus	2	0.04834023	19.65
Pycnolejeunea	14	0.0468122	22.53
Radula	200	0.05429507	16.57
Reboulia	1	0	12.37
Rectolejeunea	4	0.05112644	19.69
Riccardia	100	0.0469528	13.63
Riccia	150	0.19131466	11.08
Ricciocarpos	1	0	8.78
Riella	20	0.03739081	10.59
Saccogyna	1	0	14.36
Saccogynidium	7	0.0210732	15.12
Sauteria	2	0.03374829	5.66
Scapania	90	0.1137462	8.00
Schiffneria	1	0	13.62
Schiffneriolejeunea	15	0.14025084	16.59
Schistochila	75	0.01977709	13.29
Schistochilopsis	10	0.03299611	9.99

Schljakovianthus	1	0	-1.46
Solenostoma	120	0.1022524	12.77
Southbya	4	0.03055956	7.82
Sphaerocarpos	5	0.04049743	10.59
Sphenolobus	3	0.07022864	2.28
Spruceanthus	16	0.12024651	19.78
Stephaniella	4	0.084719	8.10
Stictolejeunea	3	0.11408071	20.59
Symbiezidium	4	0.04561849	19.69
Symphyogyna	20	0.18441763	14.86
Syzygiella	40	0.03124481	12.42
Targionia	3	0.03685395	16.35
Telaranea	30	0.05602851	16.70
Temnoma	7	0.04077524	12.91
Tetralophozia	4	0.08197146	10.32
Thysananthus	30	0.08240137	19.16
Treubia	7	0.02704477	12.27
Triandrophyllum	4	0.04233943	9.87
Trichocolea	15	0.06292149	12.43
Tritomaria	8	0.03516144	3.72
Tuyamaella	6	0.07347422	15.83
Tuzibeanthus	1	0	9.09
Wettsteinia	4	0.06106246	11.62
Wiesnerella	1	0	16.51
Xylolejeunea	5	0.0567993	23.49
Zoopsidella	5	0.03857186	13.20
Zoopsis	8	0.04287472	15.51

Table S2. Genera of our dataset which were not assignable to the time-calibrated phylogeny of Laenen et al. [37] and were thus excluded from the analyses.

Genera of our dataset unassignable to the Phylogeny of Laenen et al. [37]
Alobiella
Amphicephalozia
Anastrophyllum
Andrewsianthus
Biantheridion
Capillolejeunea
Cephaloziopsis
Chaetocolea
Chandonanthus
Chonecolea
Conoscyphus
Cryptolophocolea
Dactylophorella
Denotarisia
Dibrachiella
Endogemma
Fuscocephaloziopsis
Haesselia
"Lejeuneaceae sp."
Leptolejeunea
Lophonardia
Metalejeunea
Micropterygium
Mytilopsis
Nanomarsupella
Neesioscyphus
Neolepidozia
Pictolejeunea
Pteropsiella

Ruizanthus
Schizophyllopsis
Sphenolobopsis
Trabacellula
Tricholepidozia
Vanaea
Verdoornianthus
Vitalianthus
Zantenia

Table S3. Summary of models regressing overall and epiphytic DivElev (mean diversification rate per elevational band) and AgeElev (mean genus age per elevational band) data set without oceanic islands. Bio1, annual mean temperature (°C); Bio5, max temperature of warmest month (°C); Bio6, min temperature of coldest month (°C).

Responding variable	Coefficients	Coefficient estimate	Standard error	P value	R_p^2/ (adj.) R^2
DivElev	(Intercept)	-2.693	0.034	<0.001	
(all)	Bio1	0.022	0.002	<0.001	0.29
	(Intercept)	-1.948	0.152	<0.001	
DivElev	Bio5	-0.071	0.016	<0.001	
(all)	Bio5^2	0.002	0.000	<0.001	0.2
DivElev	(Intercept)	-2.54	0.021	<0.001	
(all)	Bio6	0.018	0.002	<0.001	0.34
	(Intercept)	50.2	2.134	<0.001	
AgeElev	Bio1	3.261	0.339	<0.001	
(all)	Bio1^2	-0.133	0.013	<0.001	0.3
	(Intercept)	17.65	7.93	<0.5	
AgeElev	Bio5	5.796	0.860	<0.001	
(all)	Bio5^2	-0.164	0.022	<0.001	0.19
	(Intercept)	67.952	1.181	<0.001	
AgeElev	Bio6	0.528	0.098	<0.001	
(all)	Bio6^2	-0.058	0.007	<0.001	0.24
	(Intercept)	-3.091	0.145	<0.001	
DivElev	Bio1	0.067	0.019	<0.001	
(Epi)	Bio1^2	-0.001	0.001	<0.5	0.41
DivElev	(Intercept)	-3.026	0.080	<0.001	
(Epi)	Bio5	0.031	0.004	<0.001	0.33
DivElev	(Intercept)	-2.593	0.033	<0.001	
(Epi)	Bio6	0.022	0.003	<0.001	0.33
AgeElev	(Intercept)	90.332	2.678	<0.001	
(Epi)	Bio1	-1.547	0.162	<0.001	0.38
AgeElev	(Intercept)	102.162	4.206	<0.001	
(Epi)	Bio5	-1.709	0.195	<0.001	0.34
AgeElev	(Intercept)	77.54	1.688	<0.001	
(Epi)	Bio6	-1.116	0.139	<0.001	0.3
